# Digital nursing: from misinformation to protagonism in public health

**DOI:** 10.17533/udea.iee.v44n1e12

**Published:** 2026-03-31

**Authors:** Neyson Pinheiro Freire, Isabel Cristina Kowal Olm Cunha, Luciano Garcia Lourenção

**Affiliations:** 1 Journalist, PhD. Email: neysonfreire@gmail.com. Corresponding author https://orcid.org/0000-0002-9038-9974 Universidade Federal de São Paulo Brazil neysonfreire@gmail.com; 2 Nurse, PhD. Associate Professor. Email: isabelcunha@unifesp.br https://orcid.org/0000-0001-6374-5665 Universidade Federal de São Paulo Brazil isabelcunha@unifesp.br; 3 Nurse, PhD. Advisor at the Ministry of Social Security, Brasília, Brazil. Email: lucianolourencao.enf@gmail.com https://orcid.org/0000-0002-1240-4702 Universidade Federal do Rio Grande at the Ministry of Social Security Brasília Brazil lucianolourencao.enf@gmail.com; 4 Escola Paulista de Enfermagem, Universidade Federal de São Paulo, São Paulo, Brazil. São Paulo Brazil; 5 Escola de Enfermagem, Universidade Federal do Rio Grande, Rio Grande, Rio Grande do Sul, Brazil. Rio Grande Rio Grande do Sul Brazil

**Keywords:** social media, community health nursing, information dissemination, infodemic, public health., redes sociales, enfermería en salud comunitaria, difusión de información, infodemia, salud pública., Mídias Sociais, Disseminação de Informação, Enfermagem em Saúde Comunitária, Infodemia, Saúde Pública.

## Abstract

**Introduction.:**

Social media has transformed health communication on a global scale, becoming an integral part of the lives of billions of people connected to the internet. More than 5.5 billion individuals worldwide already use the internet, and a large proportion of this contingent regularly accesses social media, where they often seek and receive information about health, news, and daily guidance, which broadens access but also intensifies the risks of infodemic, recognized by the World Health Organization as a threat to public health. This context opens space for nursing professionals to act as credible voices and agents of social trust. This article discusses the digital influence of nursing and advocates for its strategic role in combating misinformation and promoting health literacy.

**Content.:**

Instagram is the main platform used for educational, emotional, and motivational content. The audience expresses bonds of representation, support, and inspiration. However, the lack of regulation and the precarious nature of digital work reveal ethical and institutional vulnerabilities.

**Conclusion.:**

Digital nursing is a strategic resource for public health. Ignoring its representativeness means leaving society hostage to misinformation. It is urgent to recognize, support, and qualify this presence, integrating it into communication and health promotion policies, transforming reach into care, influence into science, and representativeness into social trust.

## Resumen

**Introducción.:**

Las redes sociales han transformado la comunicación en materia de salud a escala mundial, convirtiéndose en parte integrante de la vida de miles de millones de personas conectadas a Internet. Más de 5,5 billones de personas en todo el mundo ya utilizan Internet y una gran proporción de ellas accede regularmente a las redes sociales, donde a menudo buscan y reciben información sobre salud, noticias y orientaciones cotidianas, lo que amplía el acceso, pero también intensifica los riesgos de la infodemia, reconocida por la Organización Mundial de la Salud como una amenaza para la salud pública. Este contexto abre espacio para que los profesionales de enfermería actúen como voces de credibilidad y agentes de confianza social. Este artículo analiza la influencia digital de la enfermería y defiende su papel estratégico en la lucha contra la desinformación y la promoción de la alfabetización en salud.

**Contenido.:**

Instagram concentra la actuación, con contenidos educativos, afectivos y motivacionales. La audiencia expresa vínculos de representatividad, apoyo e inspiración. Sin embargo, la ausencia de regulación y la precariedad del trabajo digital revelan vulnerabilidades éticas e institucionales.

**Conclusión.:**

La enfermería digital es un recurso estratégico para la salud pública. Ignorar su representatividad significa dejar a la sociedad a merced de la desinformación. Es urgente reconocer, apoyar y fortalecer esta presencia, integrándola en las políticas de comunicación y promoción de la salud, transformando el alcance en cuidado, la influencia en ciencia y la representatividad en confianza social.

## Resumo

**Introdução.:**

As redes sociais transformaram a comunicação em saúde em escala global, tornando-se parte integrante da vida de bilhões de pessoas conectadas à internet. Mais de 5.5 bilhões de indivíduos em todo o mundo já utilizam a internet e uma grande proporção desse contingente acessa redes sociais regularmente, onde frequentemente buscam e recebem informações sobre saúde, notícias e orientações cotidianas, o que amplia o acesso, mas também intensifica os riscos da infodemia, reconhecida pela Organização Mundial da Saúde como uma ameaça à saúde pública. Esse contexto abre espaço para que profissionais de Enfermagem atuem como vozes de credibilidade e agentes de confiança social. Este artigo discute a influência digital da Enfermagem e defende seu papel estratégico no enfrentamento à desinformação e na promoção da literacia em saúde**.**

**Conteúdo.:**

O Instagram concentra a atuação dos profissionais, com conteúdos educativos, afetivos e motivacionais. A audiência expressa vínculos de representatividade, apoio e inspiração. Entretanto, a ausência de regulação e a precarização do trabalho digital revelam vulnerabilidades éticas e institucionais.

**Conclusão.:**

A Enfermagem digital é um recurso estratégico para a saúde pública. Ignorar sua representatividade significa deixar a sociedade refém da desinformação. É urgente reconhecer, apoiar e qualificar essa presença, integrando-a às políticas de comunicação e promoção da saúde, transformando alcance em cuidado, influência em ciência e representatividade em confiança social.

## Introduction

The COVID-19 pandemic exposed to the world something that health professionals have known for a long time: information can save lives, but it can also destroy them.^1^ Between 2020 and 2022, we witnessed an avalanche of false messages about miracle cures, supposed preventive treatments, and attacks on vaccines, which spread faster than any official protocol. This epidemic of rumors gained a name - infodemic - and transformed health communication into one of the main battlegrounds of our time.^2^ The World Health Organization has come to consider the infodemic a global risk, with a direct impact on behaviors, adherence to public policies, and trust in science. Globally, it is estimated that more than 5.5 billion individuals use the internet, routinely resorting to social networks as one of the main sources of information, including on health-related topics.^3^ Scientific evidence indicates that this massive use of digital platforms expands access to knowledge, but also intensifies the circulation of inaccurate or false content, causing the infodemic to cease being a metaphor and become a concrete public health problem, with impacts on individuals, families and health systems in different sociocultural contexts.^4^ In this context, Nursing has become a protagonist on an unexpected front: digital influence. The largest professional category in health, with a presence in all regions of the country and daily contact with the population, Nursing has also gained space on social networks.^5^ Nurses with thousands of followers have begun to occupy cell phone screens with tips, reflections, accounts of daily life and educational content. This movement gave face, voice, and emotion to the science of care-and, at the same time, revealed both the potential and the fragilities of a territory marked by the absence of regulation and the dominance of commercial algorithms.^6^ This phenomenon reflects a new dimension of care: digital presence as a symbolic and communicational extension of professional practice.

Ignoring the digital presence of Nursing is a strategic error. While doctors, research institutions, or government agencies struggle to communicate with the public in simple language, Nursing influencers conquer this space through proximity. They are the ones who receive messages from patients seeking clarification, from students with questions, or from anxious family members trying to understand guidelines on prevention and treatment. This bridge cannot be underestimated: it is a concrete form of digital care, operating beyond the traditional boundaries of the hospital and clinical practice.^7^ This is a new ecology of communication in health, where trust is built through empathy, authenticity, and continuous presence on social networks.

However, it is also necessary to recognize the risks of this scenario. The same logic that enables the connection between professionals and the public can be instrumentalized for the dissemination of misinformation. Evidence suggests that emotionally charged content, even if lacking a scientific basis, tends to reach a wider audience than official and technically sound communications, since digital platform algorithms prioritize engagement metrics-such as likes, comments, and shares-over the veracity of the content.^8^ Thus, nursing influencers operate in a minefield: while they can be strategic allies of public health, they can also, unintentionally, reinforce misinformation.^9^ Understanding this ambiguity is essential for formulating policies that transform digital influence into a tool for promoting evidence-based health.

Recent data from an empirical study reveal the extent of this phenomenon in Brazil.^10^ The study analyzed 324 profiles of digital nursing influencers, who together had more than 45 million followers. Most of these influencers operate on Instagram, with content that combines education, motivation, and humor. The target audience is predominantly young, female, and actively employed. Analysis of the interactions revealed that the established connections go beyond technical information, highlighting feelings of representation, support, admiration, and belonging. These findings indicate that the impact of digital nursing is not limited to the cognitive dimension, but also involves emotional, identity-related, and social aspects, reinforcing that it is a socio-communicative force with educational and mobilizing potential.

Given the results, the authors argued for the need for a strategic choice: whether to continue treating the digital presence of Nursing as a peripheral phenomenon, little considered in the formulation of public policies, or to recognize it as a central strategy in combating misinformation and promoting collective health. This article supports the second position. It argues that the digital representation of Nursing is not a passing phenomenon, but a necessary response to a context in which the credibility of science is constantly strained. From this perspective, digital Nursing can - and should - be strengthened as an ally in combating misinformation, building social trust, and democratizing access to health information. To this end, it is essential to address structural challenges, such as qualifying evidence-based content production, institutional support, regulation of digital work, and preparing the profession to critically engage with the algorithmic logic of platforms.

After all, it is not enough to combat fake news with isolated fact-checking initiatives. It is necessary to strengthen those who already have a voice, credibility, and connection with the population. Digital nursing represents precisely this link: professionals who transform reach into care, who give a human face to science, and who, if properly supported, can become solid barriers against the infodemic. This is, therefore, a strategic agenda for strengthening public health and scientific communication in the 21st century. Given the above, this theoretical essay aims to discuss the digital influence of nursing and defends its strategic role in combating misinformation and promoting health literacy.

### The power of digital nursing

In analyzing the performance of Brazilian nurses on social media, the first observation that emerges is quantitative: digital nursing is already a mass phenomenon. It is not a restricted set of isolated profiles, but a network with national reach and significant projection. The survey of 324 influencers in the field shows that the category has found in digital social networks not only a space for expression, but also a territory of symbolic dispute and identity strengthening.^10^ To get an idea of ​​the magnitude of this reach, it is an audience comparable to the total population of countries like Argentina or Spain, reinforcing that digital nursing operates today on a socio-communicational scale equivalent to large national collectivities. When nurses speak on Instagram, TikTok or YouTube, millions listen. This magnitude reveals a new communicational ecosystem, in which nursing occupies a central role in the circulation of health information.^11^ This massive reach is relevant not only for its quantity, but above all for the quality of the audience. Furthermore, the findings of the study that analyzed 324 profiles of digital influencers in Nursing show that the majority of followers are young adults, predominantly women, in the midst of their training or professional practice. They are mostly students aspiring to enter the career, professionals seeking to update their knowledge, and also patients and family members who recognize these profiles as an accessible, close, and reliable source of health information.^10^

This follower base is not amorphous: it is organized into bonds of belonging, admiration, and learning. In other words, there is a relationship of trust that transforms each post into an opportunity for direct impact on individual and collective behaviors.^12^ This is a contemporary form of care mediated by communication, in which the symbolic and affective bond enhances learning and adherence to health practices.^13^ This is the differentiating factor of digital Nursing compared to other health agents. While doctors or institutions often speak in a technical, distant, and hierarchical way, Nursing influencers build a language of closeness. They present themselves as colleagues, companions on the journey, or even virtual friends. This horizontality dialogues with the essence of social networks, designed to reduce barriers and eliminate intermediaries.^14^ That is why a nurse's "story" showing the reality of a shift can mobilize more empathy than an official report published on institutional websites.^7^ This form of horizontal communication reinforces the credibility built in daily interaction and makes the discourse of Nursing more accessible and legitimate in the eyes of the public.^15^

Another decisive aspect identified in the analysis of the 324 digital influencer profiles in Nursing is the plurality of approaches. The themes range from humorous content and professional life to political debates on statutory minimum wage for nurses and working conditions, including educational materials on procedures, vaccination campaigns, maternal health, aesthetics, and entrepreneurship.^10^ This diversity demonstrates that digital Nursing is not restricted to a single narrative framework, but unfolds into multiple voices that broaden the social visibility of the profession and offer society a representative mosaic of its practices and identities. At the same time, this plurality strengthens the internal representativeness of the category, since different profiles engage with distinct demands and segments, reinforcing the inclusive and democratic character of Nursing's digital presence. The netnographic analysis applied to the 324 digital influencer profiles in Nursing^10^ corroborates this diagnosis. The followers' comments recurrently reveal feelings of support, inspiration, admiration, and identification, indicating that the public not only consumes the content but also symbolically recognizes itself in it. This specific phenomenon of digital identification and representation finds support in the literature, which describes Nursing influencers as “symbolic mirrors” capable of reflecting the pains, achievements, and aspirations of a historically undervalued profession, yet central to public health.^11^ This symbolic recognition operates as a mechanism of collective valorization, strengthening professional pride and social trust in Nursing.

It is also worth highlighting the educational capacity of these profiles. Many dedicate part of their work to accessible scientific dissemination, translating technical terms into everyday language and bringing care practices closer to the daily lives of the population. In times of vaccine hesitancy, for example, a simple explanation of the side effects of a vaccine, made in a short and direct video, can be more convincing than an institutional campaign. Here is an important key: the strength of digital nursing lies in its ability to humanize science.^16^ This affective and didactic translation of scientific knowledge brings information closer to diverse audiences and broadens the reach of evidence-based health messages.^17^

There is no denying that digital nursing already exerts a significant influence on health behaviors and perceptions, configuring itself as a social capital of strategic value. This finding stems from both the analysis of 324 profiles of digital influencers in Nursing^10^ and recent literature on digital communication in health.^17^^,^^18^ However, a mismatch persists between the impact actually achieved by these professionals on social media and the institutional recognition of this activity. Despite quantitative evidence on reach, engagement and influence, the digital presence of health professionals - including Nursing - remains poorly incorporated into public policies and formal regulatory frameworks, often being ignored or underestimated by decision-making bodies.^18^ This gap highlights the need to integrate, in a more systematic way, the digital dimension of Nursing into communication and health education policies, recognizing its potential in promoting the digital and scientific literacy of the population.

The findings from the analysis of the profiles of digital influencers in Nursing^10^ highlight a relevant paradox: Nursing builds relationships of trust with millions of people on digital social networks on a daily basis, but this presence is not yet incorporated in a structured way into official health communication strategies. The literature on digital communication and misinformation in health points out that the non-integration of social actors with high credibility and communicational reach represents a loss of strategic potential and increases society's vulnerability to the circulation of inaccurate information.^17^ In light of these empirical and theoretical elements, it is argued that recognizing the strength of digital Nursing goes beyond the field of symbolic valuation or academic interest, configuring itself as an emerging public health issue. Valuing this digital protagonism implies recognizing that, in the contemporary context, care is also expressed through the production and circulation of reliable information and socially engaged communication.

### The risk of the infodemic

While the power of digital nursing is undeniable, so are its risks. The environment in which these influencers operate is precisely the same one that multiplies fake news, conspiracy theories, and miracle cures. It is therefore impossible to talk about digital protagonism in health without confronting the minefield of the infodemic. This is a phenomenon that challenges not only health communication but also professional ethics and public trust in institutions.^17^ The concept of infodemic was coined by the World Health Organization (WHO) to describe the epidemic of information - true or false - that spreads at a faster rate than society's own capacity for verification and response. According to the WHO, this phenomenon intensified critically during the COVID-19 pandemic, when the massive circulation of unverified content began to coexist with, and often compete with, official evidence-based communications. In this context, while health authorities were releasing technical bulletins and health protocols, informal content and homemade videos promoting therapies with no proven efficacy went viral.^1^ Evidence accumulated throughout the pandemic links this scenario to increased vaccine hesitancy, dangerous self-medication practices, and weakened trust in health institutions, with more severe impacts on socially vulnerable populations. The infodemic, in this sense, is not just a communication problem, but a social determinant of health, as it directly interferes with self-care practices and adherence to public policies.^19^

This infodemic scenario did not disappear with the end of the health crisis. On the contrary: it has become the standard for digital communication. Social networks reward content that generates more engagement - ​​not the most truthful content. Emotion is worth more than evidence, controversy more than consensus, catchy phrases more than scientific reviews.^17^ In this digital game, even well-intentioned health professionals can be swallowed up by the logic of the algorithm, reproducing exaggerated simplifications or information without proper checking in order not to “lose relevance”.^1^ This dynamic reveals a contemporary paradox: the search for visibility can, unintentionally, compromise the scientific integrity of the message.^16^ In the case of Nursing, this risk is even more delicate. The bond of trust established between influencer and follower transforms each post into an implicit recommendation. When a nurse with thousands of followers shares a personal account of a particular treatment or product, her followers tend to interpret it as professional guidance, even if that was not the intention.^11^ The power of influence here is not only communicational: it is clinical, behavioral, and social.^2^ In light of the profiles of digital influencers in Nursing^10^ analyzed, this dimension of influence is interpreted as indicating that the digital discourse of Nursing should be understood as part of the extended continuum of care, with potential repercussions on attitudes and decisions in health.

Another critical point identified is the absence of systematic mechanisms for verifying the digital performance of Nursing in Brazil. Unlike clinical practice, which is rigidly regulated by professional councils, communication in digital environments still lacks clear guidelines regarding ethical limits, technical responsibilities, professional identification, and minimum quality standards for the content disseminated. In the absence of these parameters, each influencer acts autonomously, guided by their individual experience, personal judgment or, in some cases, by the pursuit of greater visibility and engagement. This institutional gap weakens professional accountability - understood here as the ability to identify authors, assign ethical responsibilities and ensure transparency in the production and circulation of health information - and hinders the protection of Nursing's credibility in the digital environment.^17^ Empirical analysis shows that some of the profiles of digital influencers in Nursing^10^ do not present basic mechanisms for professional identification, such as badges or verifiable information, which raises questions about authorship, underlying interests and the legitimacy of the messages conveyed. This opacity contrasts with the principles of transparency that should guide health communication and creates an environment conducive to informational manipulation and the dissemination of pseudoscientific content.^15^

The World Health Organization warns that the infodemic is not limited to the circulation of false information, but also includes an excess of content-including evidence-based content-which, by cognitively overloading individuals, hinders the critical evaluation of information, generates confusion, and can lead to decision paralysis, especially in contexts where people follow multiple sources with divergent approaches, compromising informed health decision-making.^1^^,^^17^ This phenomenon, known as “information fatigue,” has been associated with reduced trust in science and disengagement from preventive practices, with direct implications for public health.^20^ These risks do not diminish the importance of digital nursing. On the contrary: they reinforce the need to treat it as a strategic field that requires training, support, and regulation. Pretending that the problems do not exist is to condemn professionals to improvise in a highly complex scenario and leave millions of followers exposed to misinformation. Recognizing vulnerabilities is the first step toward consolidating digital nursing as an ethical and educational force against the infodemic. What is at stake, therefore, is not whether or not nursing should be present on social media-that is already a fait accompli. The central question is how to ensure that this digital presence becomes an antidote to the infodemic and an instrument for strengthening health literacy, and not just another of its symptoms.^2^

### The strategic opportunity of digital nursing

If the infodemic represents a threat, digital nursing represents a possible response. The same environment that potentiates misinformation also offers an unprecedented chance to bring science and society closer together.^17^ By occupying this space with responsibility and technical competence, nursing reaffirms its historical vocation to educate and care in multiple contexts. It is precisely at this point that this professional category, due to its history, reach, and representativeness, can become a protagonist in promoting more accessible, empathetic, and evidence-based health communication.

It is no exaggeration to say that nursing is the health profession that most engages with the population. Whether in the hospital, the health center, or during home visits, it is nurses who explain procedures, guide families, comfort patients, and translate prescriptions into accessible language.^14^ This communicative vocation, when transported to social networks, expands and multiplies. The research data are clear: the most engaging content is precisely that which combines health education with affect, humor, and inspiration. In other words, what Nursing has been doing for centuries in face-to-face care finds a new channel in the digital environment.^21^ This transposition of face-to-face care to the digital expands the social reach of Nursing and reaffirms its role as a mediator between scientific knowledge and everyday health practices.^11^

This power of Nursing needs to be recognized as a public health strategy. Instead of viewing Nursing influencers merely as a curiosity, it is time to integrate them into institutional campaigns, form partnerships for scientific dissemination, and invest in training so that their performance is increasingly ethical and evidence-based.^21^ It should be clarified that this is not about transforming professionals into propagandists for the government, the pharmaceutical industry, or any other sphere that would take advantage of this influence, but about valuing the credibility already gained with the public and putting it at the service of health promotion.^11^ It is, therefore, about recognizing Nursing influencers as digital public health agents, capable of translating scientific knowledge into socially relevant language.

There are plenty of examples: during the pandemic, nursing profiles played a central role in explaining prevention protocols, debunking rumors about vaccines, and giving visibility to the exhausting reality of shifts.^16^ At a time of widespread distrust, these voices became a refuge of trust for millions of people.^11^ This history proves that digital nursing can be mobilized in crisis situations - and it would be a monumental mistake not to prepare this social capital for future health emergencies.^1^ By acting with empathy and credibility, these professionals have shown that digital communication can be a legitimate extension of care and health education.^11^

But it is not only in crisis scenarios that this opportunity reveals itself. Digital nursing can contribute to strengthening permanent public policies: vaccination campaigns, prevention of chronic diseases, promotion of self-care, mental health, and sexual and reproductive rights. It can also act as a vector for professional valorization, by giving visibility to historical issues such as statutory minimum wage for nurses and working conditions.^22^ When a nurse with hundreds of thousands of followers speaks about the precariousness of the profession, the topic ceases to be restricted to unions and councils and reaches society in general. This generates social and political pressure that would hardly be achieved through official statements alone.^1^ In this sense, digital influence becomes an instrument of advocacy, capable of strengthening collective identity and driving institutional transformations.^23^

Another strategic point highlighted by the analysis of the profiles of digital influencers in Nursing^10^ is the dimension of representativeness. In addition to the predominantly female presence, there is significant participation of black women and professionals from working-class backgrounds. When these nurses occupy the digital space, they do not limit themselves to disseminating health information, but begin to challenge stereotypes historically associated with the profession, give visibility to frequently invisible trajectories, and strengthen the collective self-esteem of the category. The data analyzed indicate that this symbolic impact unfolds into concrete effects, such as the motivation of students, the inspiration of new professionals, and the strengthening of the social legitimacy of Nursing as a scientific field.^10^ From this perspective, digital representation emerges as an expression of the social and political role of Nursing, which goes beyond the care dimension and projects itself as a practice of professional empowerment and cognitive justice.

In light of the analysis of the profiles of digital influencers in Nursing,^10^ transforming this communicational power of Nursing into public policy requires the adoption of structuring measures. Among these, the need for systematic training programs in scientific communication and digital ethics stands out, capable of qualifying the performance of Nursing influencers and increasing the safety and quality of the information produced. The importance of strengthening institutional support networks is also evident, through partnerships with universities, professional councils or health agencies, ensuring access to up-to-date evidence, reliable data and technical support. Finally, the results point to the relevance of formally recognizing this digital activity, which can materialize in funding opportunities, awards, or the incorporation of these professionals into national health campaigns.^17^ These measures not only qualify the digital presence but also consolidate Nursing as a protagonist in public health communication.^11^

In short, the opportunity is there: digital Nursing already exists, influences, educates, and mobilizes. As evidenced throughout our analysis, this activity is configured as a relevant space for the production and circulation of health information, with the potential to expand health literacy, strengthen social trust, and contribute to collective care. Understanding and integrating this digital presence in a structured way into health communication strategies represents a promising path for strengthening more democratic, inclusive practices aligned with scientific evidence.

### Weaknesses and challenges of digital nursing

While digital nursing represents strength and opportunity, it also faces weaknesses that cannot be ignored. Romanticizing this phenomenon would be to believe that the individual goodwill of influencers alone is sufficient to transform reach into care. The reality is more complex: digital protagonism coexists with precariousness, lack of regulation, emotional overload, and vulnerability to the power of platforms.^17^ A first challenge is the so-called algorithmic precariousness.^24^ Unlike hospitals or universities, where the rules are clear and public, on social networks the visibility criteria are defined by mutable algorithms driven by profit. An influencer can, from one day to the next, have their audience drastically reduced without explanation, simply due to changes in the platform's code. This dependence on arbitrary rules places nursing professionals in a position of extreme vulnerability. Even high-quality content can be made invisible because it does not conform to the trends dictated by the algorithm.^24^

Another critical point concerns the lack of specific regulation for digital practice. In clinical practice, Nursing follows formally established ethical and technical protocols; however, in the digital environment, there is still a lack of clear guidelines regarding the limits of dissemination, relationships with sponsors, and the distinction between personal accounts and professional guidance. This context tends to generate insecurity for both influencers and the public. As discussed by Lewandowsky et al., in the so-called "post-truth" era, the lack of explicit communication criteria and informational opacity can hinder the distinction between scientific information, personal opinion, and commercial interests, which compromises the critical evaluation of health content.^25^

The precariousness of digital work constitutes a central aspect of the performance of Nursing influencers. Empirical evidence indicates that, even with high reach, this activity rarely translates into a stable source of income, implying dependence on occasional commercial partnerships and intensification of content production routines.^11^ In the context of Nursing, this dynamic is linked to the historical overload of care work, producing an accumulation of demands that can negatively affect the mental and physical health of professionals. This condition is deepened by the structural logic of digital platforms. Studies on algorithm-mediated work demonstrate that visibility criteria are opaque, changeable, and driven by economic interests, transferring the risks of instability and loss of relevance to creators.^24^ This algorithmic precariousness intensifies occupational insecurity and emotional vulnerability in digital work. The absence of institutional support contributes to consolidating this scenario. Without structured recognition of digital nursing practice, professionals tend to work in isolation, with limited access to training, technical support to combat misinformation, and mechanisms to protect against digital violence.^11^ This combination of institutional fragility and dependence on platforms compromises the sustainability of digital work and the public credibility of nursing.

From the public's point of view, vulnerability is also relevant. Analysis of the profiles of digital influencers in Nursing ^10^ showed that some of them do not present themselves in a personal or transparent way, either due to the lack of clear identification of their responsible parties, or due to the use of generic or institutional names without verifiable links. This pattern opens space for the circulation of content of uncertain authorship that takes advantage of the social credibility associated with Nursing to promote political, commercial or ideological interests. The lack of systematic verification mechanisms in these environments exposes followers to informational risks and weakens social trust in professionals who act ethically and responsibly.

Finally, there is information overload. Even correct content, when excessive, can generate confusion. Followers who follow dozens of health profiles receive fragmented, contradictory or repetitive information.^24^ Without adequate mediation, the result is anguish, disorientation and, in some cases, discredit of science.^17^ These weaknesses do not diminish the importance of digital nursing; on the contrary, they reinforce the urgency of creating support, regulation and qualification mechanisms. No professional, however engaged, will be able to sustain the fight against misinformation alone in a hostile digital environment. The challenge is collective and institutional.^11^^,^^17^ The question that arises is direct: will we continue to leave nurses alone in this battlefield, or will we build policies that protect, strengthen and recognize them as central agents of health communication in the 21st century?

## Conclusion

Digital nursing is not a peripheral curiosity or a passing fad. It is a consolidated social and communicational phenomenon, with a direct impact on how millions of Brazilians obtain information, learn about, and relate to health. Ignoring this reality means leaving society hostage to misinformation; recognizing it, on the other hand, paves the way for an innovative strategy for promoting public health. The data is clear: hundreds of nursing influencers mobilize tens of millions of followers, building bonds of trust and representation that official bodies and institutional campaigns rarely achieve. This strength, however, coexists with serious risks, ranging from algorithmic precariousness to the absence of regulation, from work overload to a lack of institutional support. The same environment that offers visibility and influence also imposes a high price on those who dare to occupy it.

Given this, the dilemma is unequivocal: either we strengthen the digital presence of nursing as an ally in confronting the infodemic, or we hand this space over to fake news. There is no neutrality; the void left by science is quickly filled by misinformation. The proposal, therefore, is clear and urgent: to transform the digital influence of Nursing into a structured public policy. This involves offering training in scientific communication, creating mechanisms for institutional support, officially recognizing influencers as digital health agents, and developing regulatory frameworks that guarantee ethics, transparency, and sustainability in this work. More than ever, it is time to align reach and responsibility, visibility and commitment, influence and science.

Digital Nursing has already demonstrated its strength. Now, it is up to society to decide whether we will waste it or enhance it. Between the noise of fake news and evidence-based care, the choice should be obvious: support those who have earned the public's trust and transform it into health, citizenship, and life. In the 21st century, influencing with science is also a way to save lives.
